# Acute Streptococcus constellatus Pyogenic Liver Abscess Due to an Atypical Presentation of Sigmoid Diverticulitis Complicated by Pericolonic Abscess

**DOI:** 10.7759/cureus.10940

**Published:** 2020-10-14

**Authors:** Daniela Navarrete, Sachin Patil, Dima Dandachi

**Affiliations:** 1 Internal Medicine, University of Missouri, Columbia, USA; 2 Infectious Disease, University of Missouri, Columbia, USA

**Keywords:** sigmoid diverticulitis, streptococcus constellatus, pyogenic liver abscess

## Abstract

Diverticulitis is a result of diverticulum inflammation that involves protrusion of the colonic wall. It is considered to be complicated when associated with an abscess, fistula, perforation of large bowel, or obstruction. The incidence of diverticulitis increases with age, and it most commonly presents as constant abdominal pain in the left lower quadrant. We report a case of a 54-year-old male with a history of hypertension who was admitted to our hospital with systemic symptoms and right shoulder pain noted to have *Streptococcus constellatus* bacteremia and an incidental finding of a single large hepatic abscess on chest imaging. Additional imaging studies revealed the presence of acute sigmoid diverticulitis complicated by pericolonic abscess. He had no known risk factors and had not experienced any abdominal pain before admission.

A liver abscess due to *S. constellatus* is a rare complication from sigmoid diverticulitis, and there are only a few cases reported as per the PubMed medical literature review. Only four other unique cases with few or no risk factors were discovered to have a pyogenic liver abscess initially, and later source was found to be due to perforated diverticulitis. Due to this rare clinical presentation, diagnosis is often delayed leading to complications requiring surgical intervention. This can result in higher mortality. Our patient had ultrasound-guided drainage of the abscess and completed a six-week course of intravenous antibiotics (ceftriaxone and metronidazole) with successful recovery.

## Introduction

Liver abscess is one of the most common visceral abscesses seen in clinical practice [1]. While hepatic abscesses are typically symptomatic, they can be asymptomatic in rare circumstances, which may delay the diagnosis [2]. Intra-abdominal infections result in liver abscess by multiple mechanisms. Diverticulitis infrequently presents without symptoms resulting in complications such as bacteremia and pyogenic liver abscess. Intra-abdominal infections spread to the liver frequently via the biliary tract [2]. In diverticulitis, the spread is via the portal vein resulting in pylephlebitis and liver abscess. The annual incidence of pyogenic liver abscesses is 2.3 cases per 100,000 people in the United States [3]. In Taiwan, the incidence is much higher, at 17.6 cases per 100,000 due to numerous cases of *Klebsiella pneumoniae* [4]. Etiologies of pyogenic liver abscesses are mostly polymicrobial, including *K. pneumoniae*, *Escherichia coli*, *Streptococcus milleri*, and anaerobic organisms [2]. The most common organism in the United States is *K. pneumoniae* in patients under 65 years of age and *E. coli* in patients greater than 65 years of age [3]. Risk factors for liver abscess include diabetes mellitus, hepatobiliary or pancreatic disease, history of a liver transplant, chronic granulomatous disease, proton pump inhibitor use, and malignancy [5-7]. Here we present a unique case of acute sigmoid diverticulitis in a white male with no risk factors and absence of abdominal pain. This resulted in a contained diverticular perforation with bacteremia and pyogenic liver abscess due to *Streptococcus constellatus*. A review of the existing medical literature reveals this presentation to be uncommon. Most prior described case reports of liver abscess due to sigmoid diverticulitis are due to *S. anginosus* and *S. intermedius* but only a few with *S. constellatus*.

## Case presentation

**Figure 1:**
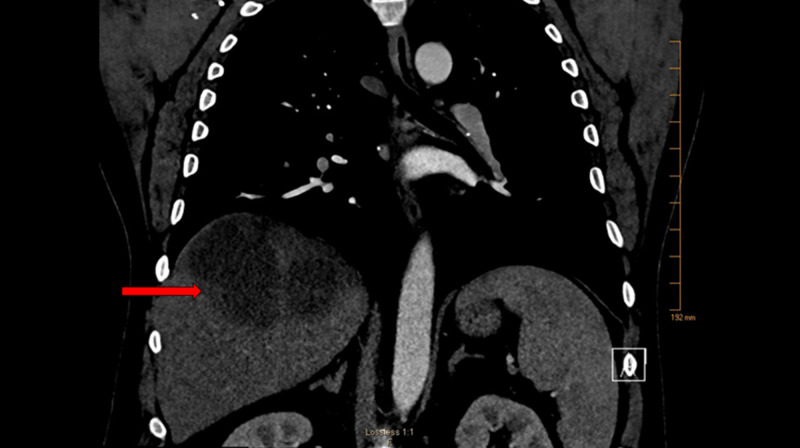
CT chest with contrast revealed large irregular loculated hypoattenuating lesion with rim enhancement in the posterior right hepatic lobe, measuring 8.0 x 12.2 x 8.8 cm, concerning for hepatic abscess

A 54-year-old white male was admitted to our hospital with four days of progressively worsening fever, chills, and right shoulder pain. Past medical history was only significant for hypertension. Approximately six weeks prior to this presentation, he began experiencing right hip pain, which radiated to his right groin. This prompted an emergency room visit where he had a computed tomography (CT) scan of his right hip that was negative for an acute abnormality. He was diagnosed with lumbar radiculopathy and treated with a muscle relaxant. Two weeks later, he had intermittent subjective fevers, chills, and hot sweats along with dyspnea prompting another emergency room visit. Chest x-ray showed right lower lobe consolidation suggestive of community-acquired bacterial pneumonia (CAP), and he was discharged on therapy with oral antibiotics. A few days after completing the oral antibiotics, he started experiencing right shoulder pain. He presented a third time to the emergency room. As the patient had dyspnea and fever, there was a concern for right-sided complicated pneumonia. A CT scan of the chest revealed a single 8 x 12 x 9 cm hepatic abscess in the absence of pneumonia (Figure 1). 

At admission, the patient was in acute distress due to rigor with a fever of 41 ºCelsius, tachypnea of 24 breaths per minute, blood pressure of 135/80 mmHg, and tachycardia of 100 beats per minute. The physical exam was remarkable for right upper quadrant tenderness on palpation. The patient had dentures with no signs of any periodontal infection. His labs were significant for leukocytosis of 18,000/mL with neutrophil predominance, lactic acid of 2.9 mmol/L, erythrocyte sedimentation rate (ESR) of 40 mm/hr, alkaline phosphatase (ALP) elevated at 164 U/L, gamma-glutamyl transpeptidase (GGT) elevated at 132 U/L, aspartate aminotransferase (AST) of 35 U/L, alanine aminotransferase (ALT) mildly elevated at 58 U/L, and normal total bilirubin. Urine analysis was negative for infection, and human immunodeficiency virus testing was negative. Hemoglobin A1c was 5.8%, which ruled out diabetes mellitus as a contributing factor. Once blood cultures were drawn, he was initiated on intravenous (IV) ceftriaxone, metronidazole, and fluids for acute sepsis due to liver abscess. He underwent ultrasound-guided drainage of the liver abscess by pigtail catheter placement. Liver abscess fluid was purulent, blood-tinged indicative of infection. Both the blood and liver abscess cultures grew *S. constellatus *susceptible to ceftriaxone, clindamycin, erythromycin, and vancomycin with intermediate susceptibility to penicillin. Repeat blood cultures done a day later were negative.

**Figure 2:**
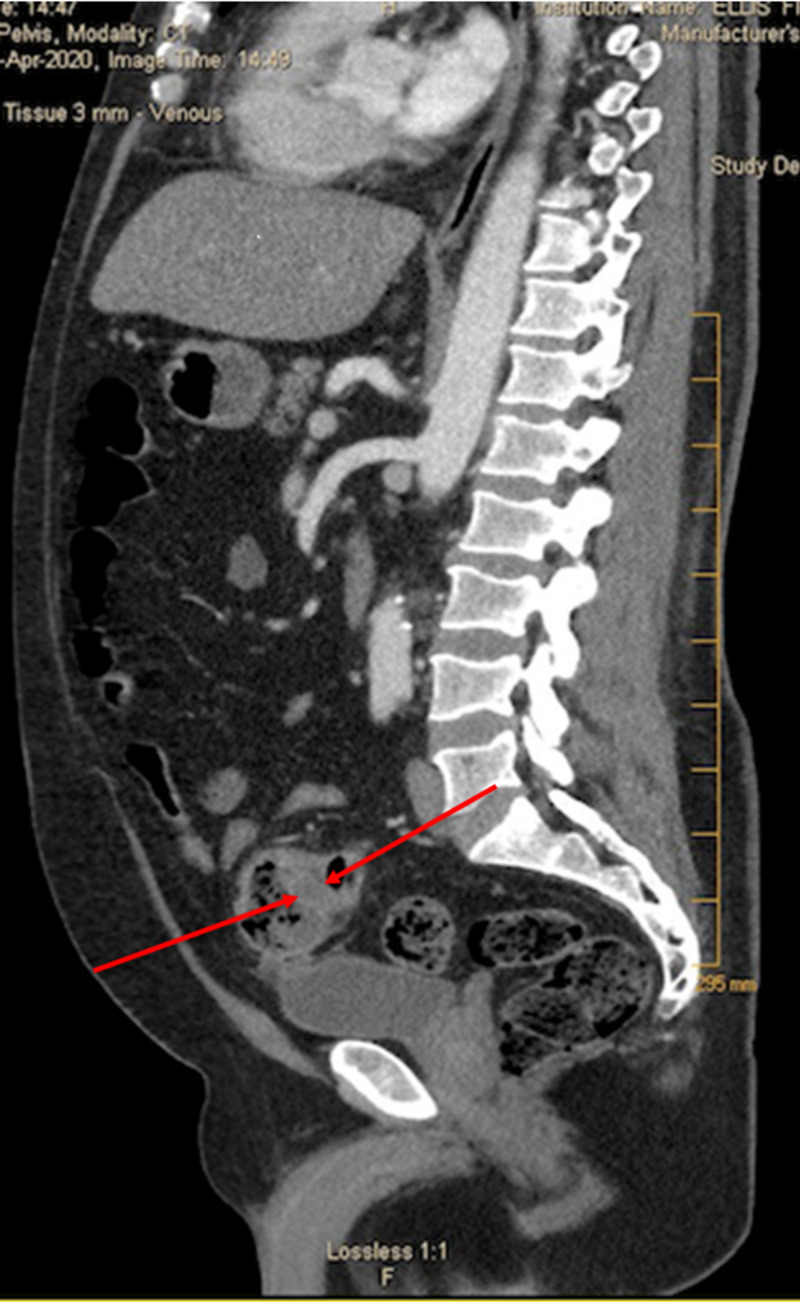
CT scan the abdomen, and pelvis revealed sigmoid diverticulitis and an adjacent small contained pericolonic abscess with dimensions of 3.5 x 4.3 x 4.1 cm.

*S. constellatus* typically originates from the oral, intestinal, and urogenital tract, but our patient had no symptoms of pathological involvement of these systems. The patient denied any abdominal pain or distension. He did admit to having loose stools three times a day on two days a week before this admission. He had no significant travel history and denied any history of gastrointestinal issues, alcohol use, or recent dental or abdominal procedures. He had a colonoscopy four years ago for screening that was normal. Due to the presence of dentures, a panorex x-ray was done, which revealed no infection. A CT scan of the abdomen and pelvis was done to identify the source, which disclosed sigmoid diverticulitis, and an adjacent 3.5 x 4.3 x 4.1 cm contained pericolonic abscess (Figure 2).

The patient showed an excellent response to the IV antibiotics, and his right shoulder pain wholly resolved. The pigtail catheter was removed on the day of his discharge. He was discharged home on IV ceftriaxone and oral metronidazole to complete a total of six weeks of IV antibiotics. A repeat CT scan of the abdomen and pelvis showed a decreased liver abscess size with a resolution of sigmoid diverticulitis and pericolonic abscess. The patient completed the antibiotics with an improvement in his symptoms. He underwent outpatient colonoscopy, which was positive for two hyperplastic descending colon polyps (removed) and sigmoid diverticulosis.

## Discussion

Pyogenic liver abscesses can be fatal if left untreated. In an indolent clinical presentation, it is essential to consider liver abscess in patients with systemic symptoms that persist despite initial treatment for presumed causes. Physicians should maintain this awareness even in immunocompetent patients without risk factors. Our patient had a delay in diagnosis due to the absence of distinct risk factors and initial physical exam findings. There are few cases reported in the medical literature related to *S. constellatus* and pyogenic liver abscess. *S. constellatus *belongs to the *S. milleri* group, which is a part of the healthy flora of the mouth, throat, colon, and urogenital tract [8]. They are associated with abscesses at multiple locations, including the brain, lung, and liver. Colonic diverticulitis was found in 27.5% of cases of pyogenic liver abscesses [9]. The pathogenesis is due to bowel perforation or leakage, leading to bacterial seeding into the hepatic artery or portal circulation [2].

A retrospective study evaluated all pyogenic liver abscess cases and found that 64% were males. That study's identifiable common risk factors were proton pump inhibitor use, diabetes mellitus, cholecystitis, cholangitis, prior liver surgery, and hepatic malignancy. Clinical features such as abdominal pain and fever were prevalent at 71% and 69%, respectively. The typical laboratory abnormalities were leukocytosis, hypoalbuminemia, elevated ALP, and GGT. About 44% of the patients had bacteremia [10]. Although our patient only had fever as a common clinical feature, he did have all the classical laboratory findings and bacteremia. A single case report details a clinical presentation with acute vision loss, acute diverticulitis, and *S. constellatus* bacteremia. This patient had no abdominal pain, but on workup was diagnosed with liver abscess and endophthalmitis. The symptoms resolved entirely with immediate antibiotic therapy [11].

These indolent presentations call to light the question of the differences in factors among those with acute colonic diverticulitis as a cause of the pyogenic liver abscess. Factors that can be considered include symptoms at presentation, patient age, race, associated microorganisms, and risk factors. Due to the CT imaging done as per our patient's initial findings, the liver abscess and bacteremia etiology were identified. In cases with no identifiable infection source, they are grouped under the class of cryptogenic pyogenic liver abscess. Approximately 40% of liver abscesses are cryptogenic [10]. A recent retrospective analysis revealed differences in factors contributing to cryptogenic and non-cryptogenic pyogenic liver abscesses. The study found that males and patients with diabetes mellitus were more likely to have a cryptogenic presentation. *K. pneumoniae* was found in 85% of the cryptogenic pyogenic liver abscess group, whereas the non-cryptogenic group had 39.5% of cases associated with this organism. *Streptococcus* species did not have a statistically significant association between the two groups. Four cases had metastatic infection, and all were patients with cryptogenic liver abscess. The most common sites of infection were the eye, brain, and lungs [12]. The medical literature review reports of an association with gastrointestinal malignancy and pyogenic liver abscesses. A case report describes a patient with *S. constellatus* septicemia complicating native mitral valve endocarditis and liver abscess associated with gastric adenocarcinoma [13]. These case reports highlight the importance of evaluating occult gastrointestinal malignancies in the absence of other identifiable sources.

Our patient had a rare atypical presentation with right hip pain radiating to his groin with no gastrointestinal symptoms. Even at the time of admission, he had right shoulder pain with no gastrointestinal symptoms. Intermittent fever with chills and sweats prompted his ED visit resulting in the additional workup, which revealed the sigmoid diverticulitis with a retained pericolonic abscess. Our patient had no identifiable risk factors for the pyogenic liver abscess. He also had a normal colonoscopy four years before this presentation. Given the significant association with occult gastrointestinal malignancy, he underwent outpatient colonoscopy, which was negative for cancer. His response to IV antibiotics and percutaneous drainage was excellent with complete recovery and a resolution was observed on imaging [14].

## Conclusions

This case highlights the rare clinical presentation of acute sigmoid diverticulitis with retained perforation. There could be differences in patients with an asymptomatic, atypical presentation, or those without risk factors. In addition, there could be a disparity in the etiology of the pyogenic liver abscess from a colonic source. It is critical to evaluate for hepatic abscess given the indolent presentation and difficulty in diagnosing as it is associated with high mortality. It is essential to rule out occult gastrointestinal malignancy in an individual greater than 50 years of age. More studies in the United States are needed to evaluate if the diverticular disease is associated with an increased risk of pyogenic liver abscess and *S. constellatus* bacteremia. Clinicians should be aware of this indolent presentation and investigate the pyogenic liver abscess source at the earliest for a better outcome.
